# Lanthanoid analysis in seawater by seaFAST-SP3™ system in off-line mode and magnetic sector high-resolution inductively coupled plasma source mass spectrometer

**DOI:** 10.1016/j.mex.2022.101625

**Published:** 2022-01-27

**Authors:** M.F. Soto-Jiménez, A.I. Martinez-Salcido, O. Morton-Bermea, M.J. Ochoa-Izaguirre

**Affiliations:** a Laboratorio de Isotopía Estable, Instituto de Ciencias del Mar y Limnología, Universidad Nacional Autónoma de México, Mazatlán, Sinaloa, México; b Posgrado en Ciencias de Mary Limnología, Universidad Nacional Autónoma de México, México; c Instituto de Geofísica, Universidad Nacional Autónoma de México, Ciudad de México 04150, México; d Facultad de Ciencias del Mar, Universidad Autónoma de Sinaloa, Paseo Claussen s/n, Apdo. Postal 610, Mazatlán, Sinaloa 82000 México

**Keywords:** Automating seawater analysis, Rare earth elements in seawater, Low-pressure ion chromatography system

## Abstract

Analysis of lanthanoids in seawater is challenging due to the complex matrix (∼35 g L^−1^ TDS) and low dissolved concentrations (in ng L^−1^). A 4-step strict analytical protocol and state-of-the-art technology were implemented and validated in this study. The 4-steps method involves the 1) sample filtration and acidification (pH<2); 2) pre-concentration by the matrix separation system, 3) off-line injection of the eluted sample; and 4) determination of lanthanoids by high-resolution inductively coupled plasma mass spectrometer (HR-ICP-MS). Since there are no certified values for lanthanoids in seawater are available, the method validation was done by analyzing SLEW-3 (estuarine water reference samples) and comparing with other reports and artificial seawater (100 ng L^−1^ lanthanoid multi-element standard solutions). SLEW-3 recovery varied from 78.6% to 106% and in artificial samples it ranged from 87 to 110%. Low recovery can be explained by complex organic in seawater, because the UV oxidation was not performed in the acidified samples. The variation was ≤10%, except for Gd, Tb, and Yb (11–13.75%). Blanks varied between 0.01 and 0.07 ng L^−1^, except for La and Ce (0.13–0.21 ng L^−1^). Blanks represent <5% SLEW-3 values and <1% synthetic seawater. The procedural detection limit varied from 0.01 to 0.03 ng L^−1^.

• Lanthanoids as geochemical tracers in seawaters
• A 4-step strict analytical protocol and state-of-the-art technology for lanthanoids analyses in seawaters
• Sample pre-concentration system for matrix separation for the detection of ultra-low lanthanoids levels

## Highlights

• Lanthanoids as geochemical tracers in seawater.
• A 4-step strict protocol and state-of-the-art technology for lanthanoids analyses in seawaters.
• Sample pre-concentration system for matrix separation for the detection of ultra-low lanthanoids levels.

## Graphical abstract

**Figure:**
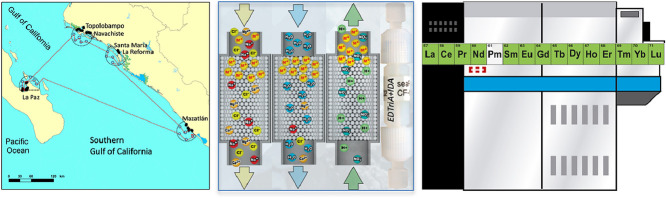

**Specifications table**

**Table:** 

Subject Area:	Environmental Science
More specific subject area:	Lanthanoids in coastal and marine ecosystems seawater
Method name:	Lanthanoid analysis in seawater
Name and reference of original method:	Analysis of rare earth elements in seawater Hathorne, E.C., Haley, B., Stichel, T., Grasse, P., Zieringer, M., Frank, M., 2012. Online preconcentration ICP‐MS analysis of rare earth elements in seawater. Geochem. Geophys. Geosystems. 13(1). Hathorne, E.C., Stichel, T., Brück, B., Frank, M., 2015. Rare earth element distribution in the Atlantic sector of the Southern Ocean: the balance between particle scavenging and vertical supply. Mar. Chem.177, 157–171. Behrens, M.K., Muratli, J., Pradoux, C., Wu, Y., Böning, P., Brumsack, H.J., Goldstein, S.L., Haley, B., Jeandel, C., Paffrath, R., Pena, L., Schnetger, B., Pahnke, K., 2016. Rapid and precise analysis of rare earth elements in small volumes of seawater-Method and intercomparison. Mar. Chem. 186, 110–120.
Resource availability:	Instrumentation A commercially available seaFAST-SP3™ system (Elemental Scientific Inc., Nebraska, USA) for matrix separation and analyte pre-concentration is used in the present method. The seaFAST-SP3™ is an ultra-clean, automated, low-pressure ion chromatography system used for undiluted seawater pre-treatment. It consists of an autosampler, a sample loop with defined volume, one pre-packed cleaning column, one pre-concentration column filled with chelating resin (hydrophilic methacrylate polymer), three 12-port valves, and four syringes. The system is capable of single-digit picogram L^−^^1^ detection limits. The 4 DX Autosampler with Dual Flowing Rinse is an ESI SC system autosampler (Elemental Scientific Inc., USA) for the complete automated sample introduction to HR-ICP-MS. This integrated autosampler increases productive instrument time by reducing sample uptake, stabilization, and rinse steps. Blank contamination at the rinse station is minimized using a gravity-fed or pressurized rinsing system, utilizing two metal-free valves to control the dual-flowing rinse solutions. Thermo Scientific™ Element XR™ High-Resolution ICP-MS (ThermoFisher Scientific Inc., Bremen, Germany) is an ultra-sensitive instrument. It is reliable multi-element analyses at trace-level concentrations, with an unequivocal separation of analyte ions from spectral interferences for the highest level of confidence. Element XR combine a dual-mode secondary electron multiplier (SEM) with a Faraday detector that increases the linear dynamic range by twelve orders of magnitude (from the of 0.2 cps to ∼5 × 10^12^ cps equivalents to sub ppq to percentages). To reduce the risk of contamination, all work on the water samples, blanks, and calibration standards was carried out in a Class 100 HEPA fume hood and the ICP-MS instrument is in a clean room with 1000 HEPA. Material and Reagents All lab materials are cleaned for trace metal analysis by submerging into 2 N HCl solution for 24 h, triple rinsing with MilliQ water, submerging into 2 N HNO_3_ solution for 24 h, and then triple rinse with MilliQ water. All reagents used in the present study are for trace analysis grade. Ultrapure HCl and HNO_3_ (J. T. Baker®, Avantor Performance Materials, LLC, Center Valley, PA., USA). High purity 21% NH_4_OH, 99.5% CH_3_COOH, and NaCl (Merck, Darmstadt, Germany). Linear calibration curves were obtained of certified standard solutions of 10 mg L^−^^1^ lanthanoids in 2% HNO_3_ (High-Purity™ Standards, Charleston, SC, USA). High-quality deionized water from the MilliQ Advantage A10 system (Millipore Corp., USA) was used in this work.

## Method details

### Step 1: Samples preparation

Seawater samples (1 L) are filtered through a 0.45 µm filter (Whatman® membrane filters nylon). Filters are replaced with plastic tweezers before a new sample is taken. For each filter sample, 1.5 ml of HNO_3_ are added to obtain pH<2, then stored into acid-cleaned 1 L Teflon bottles within 24 h after sampling. Similarly, triplicate laboratory blanks (acidified MilliQ water >18.2 MΩ) are prepared. To ensure the release of the organic species of the metal into the water and contribute to its oxidation and conversion to an inorganic form, the acidified samples and blanks are stored for a month.

### Step 2: Samples pre-concentration

**Fig. 1:**
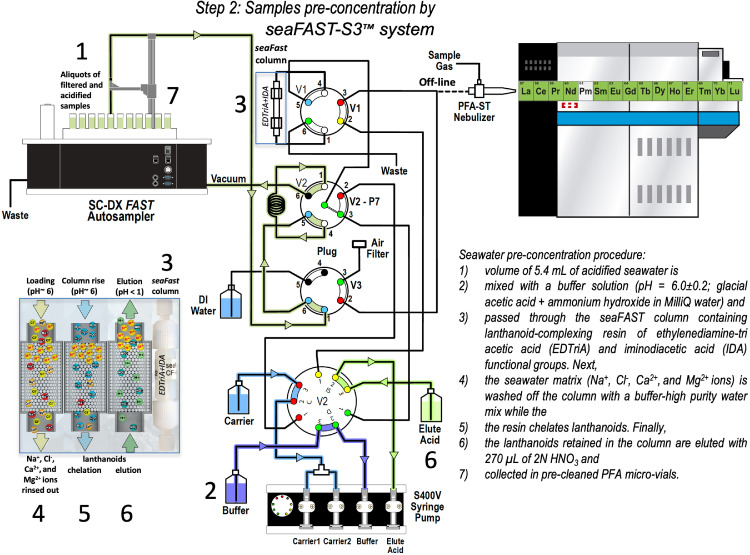
Schematic of the seawater pre-concentration procedure by seaFAST-pico™ system showing solution flow paths (Elemental Scientific Inc., USA; Behrens et al., 2016).

Different pre-concentration and matrix elimination methods have been employed to determine lanthanoids in seawater [3,^6^,11]. The seaFAST-SP3™ is a low-pressure ion chromatography system that removes the matrix and retains the lanthanoids released in low elution volumes with high concentration factors (∼20 fold). Aliquots of filtered and acidified samples are taken and transferred to 50 ml polypropylene vials for the pre-concentration step using the seaFAST-SP3™ system for matrix separation by off-line configuration. The matrix is removed and lanthanoids chelated. A schematization and brief description of the seawater pre-concentration procedure by the seaFAST- SP3™ system is provided in Fig. 1. Samples and blanks were pre-concentrated by triplicate. Instrumental operation conditions for the seawater pre-treatment seaFAST-SP3™ are shown in Table 1.

**Table 1:** Instrumental operation conditions for the seawater pre-treatment seaFAST-3™.

SeaFAST-pico	Operation conditions
Mode of analysis	Off-line
Buffer	NH_4_OH, CH_3_COOH (pH 6 ± 0.2)
Eluent	2 N HNO_3_
Sample pH	<2
Column resin	EDTriA and IDA
Initial volume of the sample	10 mL
Final volume	0.2 mL
Sample	15 min/sample
Pre-concentration factor	Up to 50

### Step 3: Off-line injection of the eluted sample

Sample's introduction into the Thermo Scientific™ Element XR™ High-Resolution ICP-MS is performed in off-line mode with an ESI SC autosampler integrated system (4 DX Autosampler with Dual Flowing Rinse). The eluted sample is introduced into the instrument through a micro-Flow PFA nebulizer (200 µL min^−1^) into the Peltier PC^3^ (Elemental Scientific Inc., USA) cooled quartz impact bead spray chamber (Fig. 1). This chamber acts as a collision-reaction cell to remove interferences that degrade the detection limits. Then, the sample is introduced into an argon plasma as aerosol droplets (nebulized).

### Step 4: Lanthanoids analysis

**Fig. 2:**
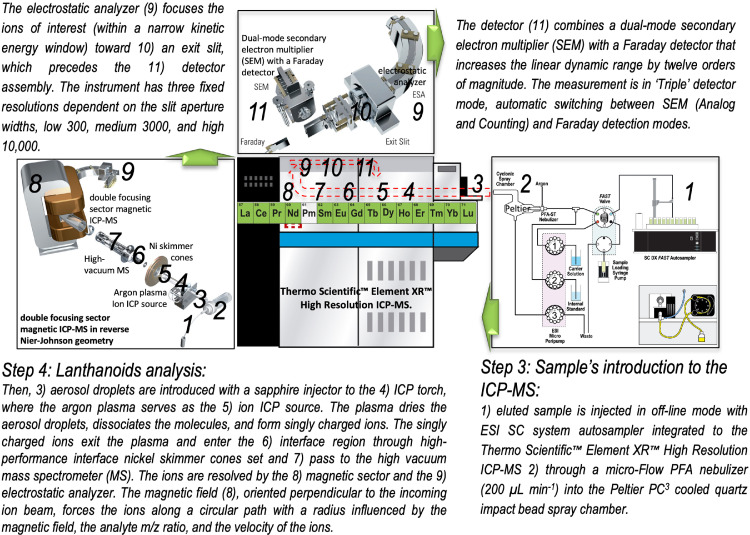
System schematic for the analyses of lanthanoids in seawater with ESI SC system autosampler integrated (4 DX Autosampler with Dual Flowing Rinse) coupled to The Thermo Scientific™Element XR™ High-Resolution ICP-MS.

The high-resolution inductively coupled plasma source mass spectrometer (HR-ICP-MS) is one of the best techniques to determine lanthanoids (La, Ce, Pr, Nd, Sm, Eu, Gd, Tb, Dy, Tb, Er, Tm, Yb, and Lu) in seawater [6]. In particular, the Thermo Scientific™ Element XR™ is a Nier-Johnson inverse geometry (e.g., focused on energy and mass/charge) dual-focus sector magnetic ICP-MS instrument that is widely used in multi-elemental determinations (e.g., lanthanoids) in complex sample matrices (e.g., seawater). A schematization and description of the lanthanoids analyses procedure by HR-ICP-MS is provided in Fig. 2. All measurements were performed in off-line mode with Thermo Scientific™ Element XR™ with an ESI SC autosampler integrated system. The measurement was conducted as ‘Triple’ detector mode, automatic switching using the Element software. Detailed instrumental conditions and the operating parameters for optimizing and ion lens voltages to achieve high and reproducible signals are provided in Tables 2 and 3. The instrument conditions were checked daily and adjusted for optimum sensitivity using a tune solution of 1 µg L^−1^ (^6^Li, ^115^In, ^238^U).

**Table 2:** Instrument Operating Conditions for the Thermo Scientific™ Element XR™ High Resolution ICP-MS.

Instrument	Operating Conditions
RF power	1500 W
Plasma Power (W)	1240
Cool gas flow (L/min)	16
Auxiliary gas flow (L/min)	0.86
Sample gas (L/min)	1.066
Spray Chamber	ESI PC3 (Peltier-cooler)
High-performance interface cone set	Nickel Cones set (ES-3000–1812 and 1802)
Mass resolution	Low
Detection mode	Triple mode: Analog, counting, Faraday
Sample per peak	20
Mass window	150
Sample time (s)	0.01
Measurement ^115^In 1 µg L^−1^	3 × 10^6^ cps (RSD= 1.79%)
Measured isotopes	^115^In, ^139^La, ^140^Ce, ^141^Pr, ^146^Nd, ^147^Sm, ^153^Eu, ^157^Gd, ^159^Tb, ^163^Dy, ^165^Ho, ^166^Er, ^169^Tm, ^172^Yb, ^175^Lu

**Table 3:** Instrument Operating Conditions for the Thermo Scientific™ Element XR™ High Resolution ICP-MS.

Instrument	Operating Conditions
Focus offset (%)	50
Ua/Ub (%)	−0.23
Extraction (V)	−2000
Focus (V)	−1049
X-deflection (V)	−2.18
Y-deflection (V)	−6.09
Shape (V)	106.5
Rotation quadrupol 1, 2 (V)	1.29, −0.87
Focus quadrupol 1, 2 (V)	−8.69, 0.7
MATSUDA-Plate (V)	150
SEM-depletion (V)	500
SEM (V)	2050
Guard Electrode	YES
Faraday deflection (V)	−177
Torch X, Y, Z-pos. (mm)	2.9, 0.1, −3.9

**Fig. 3:**
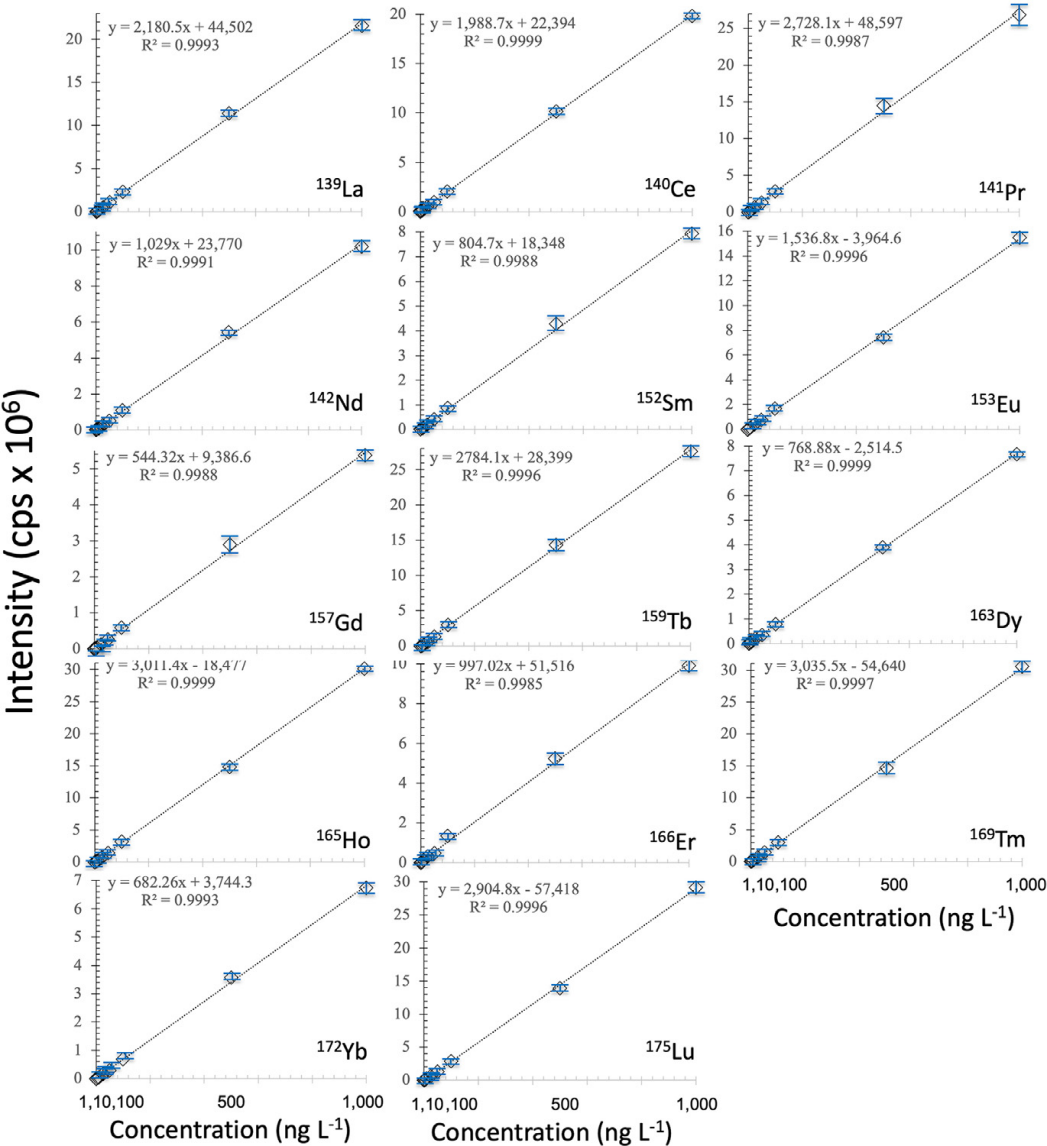
Linear calibration curves obtained of intensities (cps) measured by The Thermo Scientific™Element XR™ High-Resolution ICP-MS in six-point calibration solutions (certified standard of lanthanoids High-Purity™ Standards).

External calibration solutions of lanthanoids were prepared daily by increasing additions of a multi-element stock standard solution (10 mg L^−1^ lanthanoids in 2% HNO_3_, High-Purity™ Standard). External standardization was applied using a six-point calibration (1, 5, 10, 50, 100, and 1000 ng L^−1^, Fig. 3). Calibration solutions were processed through the SeaFAST-SP3™, such as the samples. Blank solutions of 2% HNO_3_ and NaCl matrix in 2% HNO_3_ were used as an instrumental blank, measured before each sample and standard. An internal ^115^In standard solution of 100 µg L^−1^ was prepared weekly by diluting single-element stock standard solutions. Calibration standards, reference seawater aliquots, and samples were doped with ^115^In (0.1 µg L^−1^) to monitor and correct instrumental drift. ^115^In correction was used when the instrumental drift was >5%. A Careful rinse (with 2% HNO_3_) followed by an instrumental background check was performed before every sample measurement to monitor sample to sample memory effects and correct for them, if necessary. Because of the low level of oxides formation, it was not necessary to apply mathematical correction accounting for oxide formation (e.g., UO < 2.5%). The software compares the intensities of the measured pulses to those from standards, which make up the external calibration curve, to determine the concentration of each element. Lanthanoid concentrations were calculated using the slopes of the standard external curves (R^2^ > 0.999, Fig. 3).

### Method validation

**Fig. 4:**
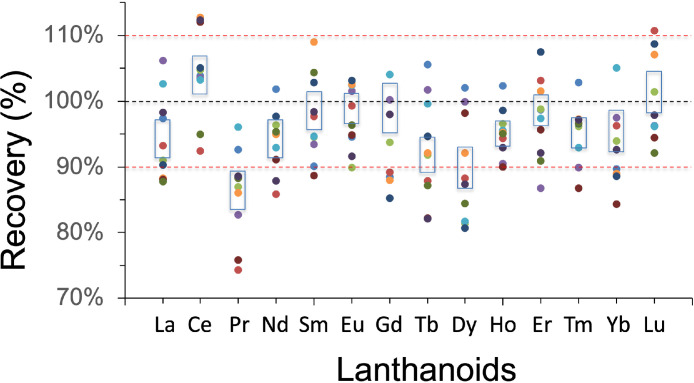
Recovery percentages of lanthanoids examined in artificial seawater sample (NaCl matrix, 35 g L^−1^) with 100 ng L^−1^ lanthanoid multi-element standard solutions (*n* = 10) measured by seaFAST-SP3™ system in off-line mode and magnetic sector high-resolution inductively coupled plasma mass spectrometer.

To check the validity of this method, we analyzed estuarine water reference samples (SLEW-3, National Research Council of Canada). Although certified values for lanthanoids are not available for these certified reference materials (CRM), we compared our results with other studies (Table 4; [1,^2^,^10^,12]). The recovery based on the SLEW-3, averaged from *78.6% for Pr to 106% for Ce.* Because UV oxidation was not performed in the pre-treatment of the acidified samples, to oxidize natural organic ligands and to dissociate lanthanoids of ligands [5], a low recovery for several elements can be explained by the presence of complex organic in seawater. Also, the external accuracy was assessed via repeat analyses of an artificial seawater sample (NaCl matrix, 35 g L^−1^). Column recovery was evaluated using 100 ng L^−1^ multi-element lanthanoid standard solutions, and the artificial sample means recoveries ranged from 95 to 100% (Fig. 4). Exceptions were observed for Ce and Lu (recovery 105–110%) and Pr and Dy (recovery 87–90%). The coefficient of variation (CV, %) for every lanthanoid in samples was ≤10% (10 repeated analyses of the artificial seawater sample), except for Gd, Tb and Yb (11–13.75%) (Table 3). The blanks varied between 0.01 and 0.07 ng L^−1^. Blanks represent <5% of the SLEW- values and <1% in the synthetic seawater. The procedural detection limit, which was calculated three times the standard deviation of 10 blank measurements, varied from 0.01 to 0.03 ng L^−1^ (Table 4).

**Table 4:** Measurements of lanthanoids concentrations (ng L^−1^) in SLEW-3: Estuarine water reference material for trace metals, Certified reference material, National Research Council of Canada (NRC – CNRC). Values are for mean ± standard deviation.

	This study	Recovery (%)^a^	Lawrence and Kamber [10]^b^	Balaram et al. [2]^b^	Arslan et al. [1]^c^	Zhu and Zheng [12]^d^
La	6.881 ± 0.476	83.8	8.22 ± 0.25	7.6 ± 0.8	7.89	8.36^b^
Ce	7.624 ± 0.503	106	7.19 ± 0.45	8.2 ± 1	7.56	7.14
Pr	1.289 ± 0.102	78.6	1.64 ± 0.03	1.7 ± 0.02	1.71	2.03
Nd	7.884 ± 0.394	98.9	7.97 ± 0.19	8.4 ± 0.5	8.42	8.37
Sm	6.425 ± 0.426	87	7.38 ± 0.21	6.5 ± 0.5	6.99	7.08
Eu	0.507 ± 0.025	92.2	0.55 ± 0.02	0.61 ± 0.04	0.6	0.68
Gd	2.83 ± 0.386	88.4	3.2 ± 0.06	2.6 ± 0.3	3.08	3.06
Tb	0.378 ± 0.052	87.9	0.43 ± 0.02	0.43 ± 0.02	0.45	0.46
Dy	2.816 ± 0.274	84.6	3.33 ± 0.08	3 ± 0.2	3.35	3.72
Ho	0.818 ± 0.058	89.9	0.91 ± 0.07	0.65 ± 0.1	0.91	0.99
Er	2.236 ± 0.142	80.4	2.78 ± 0.05	2.2 ± 0.2	2.72	2.99
Tm	0.284 ± 0.014	81.1	0.35 ± 0.01	0.65 ± 0.04	0.37	0.46
Yb	1.876 ± 0.209	101.4	1.85 ± 0.06	0.92 ± 0.07	2.05	2.77
Lu	0.288 ± 0.029	96	0.3 ± 0.01	0.32 ± 0.05	0.33	0.43

Detection limits for La, Eu, Gd, Tb, Ho, Er, Tm, Yb, and Lu 0.01, and for Ce, Pr, Dy 0.02, and for Nd and Sm 0.03 ng L^−1^

a According to reference values in Lawrence and Kember [10].

b Lanthanoids extracted by triple chelation using HDEHP (phosphoric acid 2-ethylhexyl ester -mono and di ester mixture) in heptane and measured by ICP-MS and or HR-ICP-MS.

c Lanthanoids extracted by triethylamine-assisted Mg(OH)_2_ coprecipitation/preconcentration and measured by ICP-MS.

d Lanthanoids extracted by Toyopearl AF Chelate 650M® resin and measured by FI-ICP-MS.

## ncited references

[4,[7], [8], [9]].

## Declaration of Competing Interest

The authors declare that they have no known competing financial interests or personal relationships that could have influenced the work reported in this paper.
